# Low Body Weight Predicted Bradycardia and Desaturation in Retinopathy of Prematurity Surgeries: A Retrospective Cohort Study

**DOI:** 10.3389/fped.2020.00226

**Published:** 2020-05-05

**Authors:** Bailin Jiang, Lan Yao, Hong Zhao, Jianhong Liang, Yi Feng

**Affiliations:** ^1^Department of Anesthesiology, Peking University People's Hospital, Beijing, China; ^2^Department of Anesthesiology, Peking University People's Hospital, Beijing, China; ^3^Department of Ophthalmology, Peking University People's Hospital, Beijing, China

**Keywords:** preterm, retinopathy of prematurity, postoperative apnea of the preterm infant, general anesthesia, perioperative care

## Abstract

**Background:** As a leading cause of childhood blindness, the epidemic of retinopathy of prematurity (ROP) in China is characterized by advanced stage of ROP in more mature infants than those in the West. More advanced stage of disease necessitates more complicated surgical procedures and consequently exposure to general anesthesia. These ex-prematurely born infants are at risk of developing desaturation especially after surgery under general anesthesia. Physical status, anesthetic management and surgical profile are three main facets of perioperative setting and need to be investigated to identify useful predictors for perioperative adverse events in this population of fragile infants.

**Methods:** In this retrospective cohort study, we enrolled all infants undergoing ROP surgeries at Peking University People's Hospital, Beijing, China from November 1, 2016 to October 31, 2017. Physical status, anesthesia and surgical management were analyzed by exploratory factor analysis and component matrix to explore risk factors for adverse events.

**Results:** During the 12 months, 267 cases were included, among whom 61 infants underwent two surgeries required by their ophthalmological conditions. The median postconceptual age at the time of surgery was 46 (40, 53) weeks, and median body weight was 4.0 (3.0, 6.5) kg. None of the infants was dependent on caffeine, oxygen or ventilator before surgery. Bradycardia (29/267, 10.9%) and postoperative desaturation (34/267, 13.4%) were identified as major cardiac and respiratory adverse events. Preoperative atropine, intubation and bigger body weight would prevent patients suffering from bradycardia. Infants with a body weight less than 3.15 kg had a significantly higher chance of desaturation and neonatal intensive care unit admission after ROP surgeries than those who weighed more than 3.15 kg (27.8 vs. 5.1%, OR 5.46 (95% CI 2.66-11.21), *P* = 0.000).

**Conclusion:** This study found that preoperative atropine and intubation would prevent bradycardia and low body weight was a predictor for both bradycardia and postoperative desaturation in preterm infants undergoing ROP surgeries.

## Introduction

Retinopathy of prematurity (ROP) is a leading cause of childhood blindness worldwide, which occurs only in premature infants (1). It was first described in 1940s as a complete retinal detachment behind the lens, which was the first wave of ROP due to supplement of oxygen in the incubator. ROP is characterized by an arrest of retinal vascularization in phase 1 and vasoproliferation in phase 2. There are several strategies to prevent ROP, such as maintaining low oxygen saturation (<96%) in the first few weeks after birth and high oxygen saturation (94–99%) at postconceptual age of 32 weeks (2), improving nutrition, and normalizing essential factors including insulin-like growth factor and ω-3 polyunsaturated fatty acid (3).

Early bedside screening and timely intervention are crucial to identify ROP and prevent blindness. Standardized treatment recommendations are summarized in the International Classification of Retinopathy of Prematurity first established in 1984 (4) and updated in 2005 (5). In the context of high-quality neonatal intensive care with existing criteria, only about 5–10% of infants screened will need treatment (6). Since advanced stage of ROP is seldom seen in the industrialized world, only minor surgeries will be considered, ranging from intravitreal injection of anti-vascular endothelial growth factor (VEGF) agent to transpupillary laser treatment (3).

The epidemic of ROP in China is characterized by advanced stage of ROP in more mature infants than those in the West due to the unevenly distributed medical resources, even though the National ROP screening guideline was established in 2012 (7). Children suffering from complete retinal detachment (ROP stage 5) constitute 50% of severe ROP, which required vitreoretinal surgery, the most complicated eye surgery (8). Actually prematurity is often related with underdevelopment of various systems and prone to suffer from high morbidity and mortality at surgery, and is the strongest risk factor for desaturation among infants undergoing hernia repair (9). Perioperative cardiac and respiratory adverse events remain major pediatric morbidity and mortality for children less than 1 year old (10, 11). Although infants with postconceptual age less than 46 weeks are advised to be transferred to neonatal intensive care unit (NICU) after general anesthesia (12), it is not practical with large caseloads of prematurely born infants and limited ICU beds in developing countries. Reducing the risk of cardiac and respiratory adverse events and identifying infants at risk may make full use of limited medical resources.

In this study, we aimed to identify the risk factors for major cardiac and respiratory events related with ROP surgeries in preterm infants. Specifically, we studied 206 preterm infant patients who received 267 surgeries for stage 3-5 ROP to explore possible risk factors involved in physical status, anesthetic management and surgical profile.

## Methods

We conducted a retrospective cohort study on preterm infants with ROP of stage 3-5 who received ophthalmological surgeries under general anesthesia. Anesthesia records and medical charts would be reviewed to collect demographic, surgical, anesthetic features, and cardiac and respiratory adverse events associated with surgery. We analyzed the association between these features and the adverse events to reveal potential risk factors for each adverse event. The study was approved by the Peking University People's Hospital Institutional Review Board, and parental consent was waived because of the retrospective design.

### Study Population

We retrospectively included all infants who underwent ophthalmological procedures for ROP at Peking University People's Hospital from November 1, 2016 to October 31, 2017. The majority of them were referred to our hospital where the National Pediatric Eye Center locates. Infants were excluded if they had a recent history (up to 2 weeks) of upper airway infection.

We collected information regarding their demographic, anesthetic and surgical features. Gestation age at birth and postconceptual age (the sum of the gestational and postnatal ages) at the time of surgery would be recorded. Body weight was documented in the Anesthesia Information Management System, acquired by asking the patients' parents and if any uncertainty existed the infant could be weighed on a scale in the operating room before surgery. ROP stage was estimated through the outpatient examination and confirmed and readjusted after the surgery if necessary. A preoperative history of apnea would also be collected.

### Anesthetic and Surgical Management

All patients were admitted to hospital on the day of surgery. All parents were instructed on applying topical anesthetic and mydriatic to their children preoperatively. Upon arrival at the operating room, intravenous cannula was inserted and an intravenous infusion of 2–4 ml·kg^−1^·h^−1^ ringer lactate was initiated and maintained during the surgery. Then standard monitor was established including electrocardiogram (ECG), non-invasive blood pressure and pulse saturation (SpO_2_).

General anesthesia was induced either by intravenous propofol or inhalational sevoflurane. Airway management device could be either endotracheal tube or a face mask. If an endotracheal tube was used, pressure controlled ventilation would be adopted, inspiratory pressure set as 20 cmH_2_O, and respiratory rate adjusted according to a target end tidal carbon dioxide of 35–45 cmH_2_O. Retrobulbar block with 0.5% ropivacaine 0.1 mL/kg was administered to all patients by an experienced ophthalmologic surgeon except for those undergoing laser surgery. Appropriate depth of anesthesia was ensured by central position of pupils (13). Use of atropine, opioids, muscle relaxant, or steroids was left to the discretion of the anesthesiologist in charge. After surgery, all intubated infants would be extubated in the operating room and monitored in the ward for 24 h. Infants with postconceptual age less than 46 weeks are treated as newborns, who are usually required be transferred to NICU after general anesthesia due to birth defects and patients with special conditions (12). However, with large caseloads of prematurely born infants and limited NICU beds, it is necessary to determine the NICU transfer criteria after surgery, other than simply following the postconceptual age of 46 weeks admission rule. In our center, only infant who had recurrent desaturation (<90%) and could not be relieved by oxygen supply through a face mask would be admitted to ICU for further monitoring and treatment.

Laser coagulopathy or retinal cryotherapy was given to infants suffering from ROP stage 3 or threshold disease. Scleral buckling would be applied to ROP stage 4a patients. Vitreoretinal surgery with/without lensectomy would be applied to patients suffering from ROP stage 4b and 5.

### Outcome Measurement

All episodes of bradycardia (HR <100 bpm), laryngospasm, bronchospasm, airway obstruction, apnea, and desaturation (SpO_2_ <90%) were reported as perioperative cardiac and respiratory adverse events. Bradycardia caused by oculocardiac reflex (OCR) was identified. Usually OCR was caused by pressing the globe or manipulation of extraocular muscles, which could often be corrected by cessation of surgical stimulus, or, if it did not work, atropine 0.01 mg/kg should be administered. When surgical procedures were finished and airway management tools removed, all children who had recurrent desaturation (<90%) and could not be relieved by oxygen supply through a face mask would be admitted to intensive care unit for further monitoring and treatment. Apnea was defined as a pause in breathing >10 s or a pause >5 s if associated with oxygen saturation <90% or bradycardia (Heart rate <100 bpm). This definition was adapted from GAS study (9). Pulse oximetry would be monitored for infants transferred to surgical ward, who would be discharged home 24 h after surgery, and those transferred to ICU would receive monitoring for at least 24 h also.

### Statistical Analysis

Statistical analysis was done with SPSS (version 20.0). We did univariate analysis with the student's *t*-test or Mann-Whitney *U*-test for continuous variables and the χ^2^ test for categorical variables. For all analyses, we used two-sided tests, with *P* values less than 0.05 denoting statistical significance.

In order to avoid problems of multicollinearity, exploratory factor analysis was used to reduce dimension of the confounders and to find the underlying factors with clinical significance which could extract at least 70% of squared loadings from the initial components cumulatively. Scores of those underlying factors were saved as the new variables to be used in the multivariate logistic regression models with other uncorrelated covariates. The method of backward stepwise (Likelihood Ratio), which meant variables remained in the model if they improved the model fit with the likelihood ratio test, was used in the logistic regressions models to result in selected factors.

If any underlying factor from the factor analysis was selected, all of its main components, which extracted more than 50% of loadings in the rotated component matrix, were used as selected covariates. These selected covariates would construct a new logistic regression model with other selected initial confounders. Thus by using the method of backward stepwise (Likelihood Ratio), ORs of the observable factors could be calculated. Hosmer-Lemeshow statistic and Area Under the ROC Curve (AUC) were used to measure the calibration and discrimination of the logistic regression model, and the outliers, whose absolute value of standardized residuals were greater than 2.3, were excluded from the final analysis.

In order to facilitate clinical application of our research findings, we converted the continuous variables, which are predictors for various adverse events, to binary variables. Therefore we could figure out the cut-off point for different risk variables, through Decision Tree Analysis, i.e., Classification and Regression Trees.

## Results

During the 12 months, 206 infants (median postconceptual age being 46 weeks) undergoing 267 surgeries were included in this study. Sixty-one children received a second ophthalmological procedure as needed. None of the infants was dependent on caffeine, oxygen or ventilator before surgery.

The median postconceptual age at the time of surgery was 46 (40, 53) weeks, and median body weight was 4.0 (3.0, 6.5) kg. One hundred and ninety-three (72.3%) patients were electively intubated as part of the anesthesia plan. Vitreoretinal surgery was required for 221 (82.8%) patients. Duration of surgery averaged 65.3 ± 28.0 min, whereas the duration of anesthesia was 114.7 ± 43.9 min. Difficulty in initiating venous access and observation after extubation both contributed to the gap between anesthesia duration and surgery duration (Table 1). All infants were extubated in the operating room.

**Table 1:** Demographics and measurement outcomes.

**Items**	**Data**
**Demographic features**	
Male (*n*)	189 (70.8%)
Post conceptual age (weeks)	46 (40, 53)
Gestation age at birth (weeks)	30 (29, 32)
Body weight at surgery (kg)	4.0 (3.0, 6.5)
Body weight at birth (kg)	1.3 (1.0, 2.0)
Hemoglobin concentration (g/L)	106.6 ± 17.7
**Surgical features**	
Laser/Cryotherapy (n)	22 (8.2%)
Scleral buckling (n)	24 (9.0%)
Vitrectomy with/without lensectomy (n)	221 (82.8%)
Infants received two surgeries during the study (n)	61 (22.8%)
Duration of surgery (min)	65.3 ± 28.0
**Anesthetic features**	
Duration of anesthesia (min)	114.7 ± 43.9
Number of patients with preoperative atropine	195 (73.0%)
Number of patients with preoperative steroid	183 (68.5%)
Number of patients with opioids	25 (9.3%)
Number of patients with muscle relaxant	144 (53.9%)
Number of patients receiving Intubation	193 (72.3%)
Number of patients receiving Intravenous induction	130 (48.7%)
**Cardiac and respiratory adverse events**	
Number of patients suffering from Intraoperative bradycardia	29 (10.9%)
Number of patients admitted to the NICU	34 (12.7%)

*Data were shown by n (%), Median (interquartile range) or Mean±Standard Deviation. NICU, Neonatal Intensive Care Unit*.

There was no death or cardiac arrest during the study period. As for cardiac and respiratory complications, 29 (10.9%) children suffered from intraoperative bradycardia, two of which were due to OCR and all bradycardia was resolved with intravenous atropine. After surgery, when airway management tools removed, 34 (12.7%) cases had recurrent desaturation (<90%), which could not be relieved by oxygen supply through a face mask, and were admitted to intensive care unit for further monitoring and treatment, among whom 3 cases suffered from laryngospasm after extubation. Thirty-one (11.6%) patients suffered from apnea (defined as a pause in breathing >10 s or a pause >5 s if associated with oxygen saturation <90% or bradycardia [HR <100 bpm)] (Table 1). These infants recovered inadvertently and no intubation was required during their NICU stay.

### Exploratory Factor Analysis

All variables, among which potential collinearity was considered, were analyzed with factor analysis. Five underlying factors were found: anesthesia management (preoperative atropine and steroid, muscle relaxant, airway management device, and intravenous/inhalational induction), current status of growth (postconceptual age and body weight at surgery), status of birth (gestation age and body weight at birth), duration of surgery, and current physical status (hemoglobin concentration).

### Risk Assessment of Adverse Events

Intraoperative bradycardia, occurred in 29 (10.9%) infants. All bradycardia was resolved with intravenous atropine. In multivariate analysis, anesthesia management [OR: 0.273(0.174-0.429)], current status of growth [OR: 0.295(0.154-0.565)], and duration of surgery [OR: 1.759(1.131-2.735)] were selected for constructing further logistic regression models. After 6 outliers were excluded, preoperative atropine, intubation and bigger body weight would prevent patients suffering from bradycardia (Table 2). The final logistic regression model exhibited excellent discrimination and acceptable calibration. The AUC was 0.961 (0.937-0.984), and the Hosmer-Lemeshow statistic was 0.629 (*P* = 1.000).

**Table 2:** Risk factors associated with intraoperative bradycardia.

	**Univariate (*****n*** **=** **267)**					**Multivariate (*****n*** **=** **261)**	
	**Yes**		**No**		***P* value**	**OR (95%CI)**	***P* value**
	**Total**	**Value**	**Total**	**Value**			
Atropine	195	8 (4.1%)	72	21 (29.2%)	0.000	0.125 (0.016-0.983)	0.048
Steroid	183	8 (4.4%)	84	21 (25.0%)	0.000		
Muscle relaxant	144	4 (2.8%)	123	25 (20.3%)	0.000		
Intubation	193	8 (4.1%)	74	21 (28.4%)	0.000	0.032 (0.003-0.333)	0.004
Post conceptual age (weeks)	39 (37, 43)		48 (41, 55)		0.000		
Body weight (kg)	2.0 (2.0, 3.0)		5.0 (3.0, 7.0)		0.000	0.352 (0.174-0.711)	0.004
Hemoglobin concentration (g/L)	100.6 ± 16.6		107.3 ± 17.8		0.053		
Duration of surgery (min)	70.7 ± 26.2		64.6 ± 28.2		0.274		
Duration of anesthesia (min)	115.0 ± 42.3		114.6 ± 44.2		0.962	1.029 (1.012-1.046)	0.001

For convenience of clinical application of our research findings, decision tree analysis was adopted, which revealed that 2.1 kg bodyweight at surgery was the cut-off point for bradycardia. When body weight ≤ 2.1 kg, the incidence of Bradycardia was 32.8%. When body weight was between 2.1 and 4.8 kg, Bradycardia incidence was 10.5%. When body weight > 4.8 kg, only 0.8% of infants would suffer from Bradycardia.

**Figure 1:**
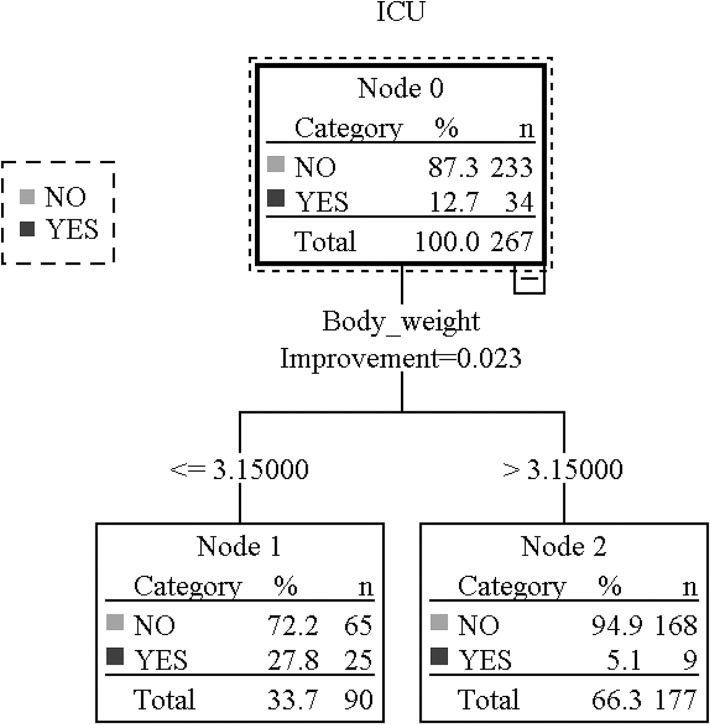
Body weight cut-off point for ICU admittance. This figure shows the decision tree of ICU admittance. Node 0 means ICU admittance of all patients, 34 being admitted to ICU after surgery and 233 not. Through Classification and Regression trees approaches, body weight at surgery was identified as an independent risk factor. When body weight ≤ 3.15 kg, the incidence ICU admittance was 25/90 (27.8%),but when[[Inline Image]] body weight>3.15 kg, the incidence was 9/177 (5.1%). infant who had recurrent desaturation (<90%) and could not be relieved by oxygen supply through a face mask would be admitted to ICU for further monitoring and treatment.

For postoperative desaturation, Anesthesia management [OR: 0.606(0.421-0.874)], current status of growth [OR: 0.268(0.150-0.479)], status of birth [OR: 0.611(0.373-1.003)], and current physical status [OR: 0.561(0.410-0.767)] were selected for further regression. After 5 outliers were excluded, the significant protective factor was body weight at surgery (Table 3). The AUC was 0.900 (0.859-0.940), and the Hosmer-Lemeshow statistic was 3.774 (*P* = 0.877). For convenience of clinical application of our research results, decision tree analysis was adopted, which revealed, for those who are lighter than 3.15 kg, the chance of ICU admittance was significantly higher than who are heavier than 3.15 kg (27.8 vs. 5.1%, *P* = 0.000) (Figure 1, Table 4).

**Table 3:** Risk factors associated with ICU admittance.

	**Univariate (*****n*** **=** **267)**					**Multivariate (*****n*** **=** **262)**	
	**Yes**		**No**		***P* value**	**OR (95%CI)**	***P* value**
	**Total**	**Value**	**Total**	**Value**			
Atropine	195	18 (9.2%)	72	16 (22.2%)	0.005		
Steroid	183	19 (10.4%)	84	15 (17.9%)	0.089		
Muscle relaxant	144	9 (6.2%)	123	25 (20.3%)	0.001		
Intubation	193	19 (9.8%)	74	15 (20.3%)	0.022		
Intravenous induction	130	8 (6.2%)	137	26 (19.0%)	0.002		
Hemoglobin concentration (g/L)	95.6 ± 15.5		108.2 ± 17.5		0.000		
Gestation age at birth (weeks)	30 (28, 32)		30 (29, 32)		0.290		
Post conceptual age (weeks)	40 (37, 42)		48 (41, 56)		0.000		
Body weight at birth (kg)	1.0 (1.0, 2.0)		1.4 (1.0, 2.0)		0.191		
Body weight at surgery (kg)	2.0 (2.0, 4.0)		5.0 (3.0, 7.3)		0.000	0.330 (0.195-0.558)	0.000

**Table 4:** Cut-off body weight for Bradycardia and ICU admittance.

	**Total**	**Number (%)**	**OR (95%CI)**	***P* value**
**Bradycardia**				
Body weight ≤ 2.1 kg	58	19 (32.8%)	6.85 (3.37-13.90)	0.000
Body weight > 2.1 kg	209	10 (4.8%)	1.00	
**ICU admittance**				
Body weight ≤ 3.15 kg	90	25 (27.8%)	5.46 (2.66-11.21)	0.000
Body weight > 3.15 kg	177	9 (5.1%)	1.00	

## Discussion

In this retrospective cohort study, bradycardia (10.9%), and postoperative desaturation (13.4%) were identified as major cardiac and respiratory adverse events in 267 cases of ROP surgical treatment. Risk factors analysis revealed that, preoperative atropine and intubation were protectors against bradycardia. And lower body weight is a predictor for bradycardia, postoperative desaturation and consequential ICU admittance other than postconceptual age.

Anesthesia management varies between developed and developing countries due to different screening program and epidemic of ROP. In developed countries, bedside ROP screening is performed under oral sucrose or anesthetic eye drops (14). Also in industrialized world, intravitrel injection of anti-VEGF agent, laser therapy and cryotherapy could be accomplished in NICU, anesthetic choices ranging from intravenous sedation to general anesthesia (15, 16). However, laser treatment was performed under sevoflurane inhalation with spontaneous ventilation through a face mask in the operating room, as previously described in a developing country (17). All intraoperative apneic episodes were resolved by cessation of surgical procedure and bag-mask ventilation in our study. For more complicated vitreoretianl surgery, retrobulbar block could be a useful adjunct for analgesia (18).

In our ex-preterm infants only study, cardiac and respiratory complications remained the major adverse events, which was similar to an observational study involving 24,165 cases of pediatric anesthesia (the incidence being 12.5 and 53% respectively) (10). Infants who received preoperative atropine and intubation had less episodes of bradycardia (HR <100 bpm), which supported the use of atropine to conquer possible OCR. With the prolongation of anesthesia duration, the prevalence of bradycardia increased. It could be postulated that for longer surgeries and anesthesia, accumulation of anesthetic might inhibit cardiovascular system and the effect of atropine faded as time lapsed. Meanwhile a bigger body weight protected the infants from bradycardia, which could be explained by the fact that a normally growing rate of body weight is the reflection of general good health condition (19). For infants who were <2.1 kg, shortening surgery duration and preventive atropine should be considered to preclude bradycardia. Patients with a recent history of upper airway infection (*n* = 5, data not shown) was not included in our study, but were safely managed by intravenous induction and use of laryngeal mask airway in accordance with a newly implemented guideline (20).

As for postoperative apnea, a smaller body weight is a strong indicator instead of postconceptual age. Infants with a body weight less than 3.15 kg have a significantly higher chance of desaturation and ICU admittance after ophthalmological surgeries than those who weighed more than 3.15 kg (27.8 vs. 5.1%, *P* = 0.000). In this study, there were 25 infants of the same postconceptual age, i.e., 37 weeks, whereas their body weight ranged from 1.4 to 4 kg (data not shown). Preterm infants are born during the fastest human growth period, whose body weight is expected to grow six-fold between gestation 22 to 40 weeks. Actually optimal growth of preterm infants is considered to be equivalent to intrauterine rates (19). However in developing countries, there are more constrains influencing infants' growth. A close to normally born body weight is a good indicator for general well-being and less episodes of postoperative apnea, which was confirmed by our study. Analyzed from 8 prospective studies, Cote found that the overall risk of apnea in patients less than postconceptual age 48 weeks is 5%, and this risk does not decrease to <1% until patients reach postconceptual age of 54 weeks after inguinal herniorrhaphy (21).

Based on the findings of our study, it could be concluded that we need to take both body weight and postconceptual age into consideration when we apply anesthesia to these premature infants, and the transfer to ICU after surgery. The infants are usually not covered by medical insurance, so it is necessary to avoid extra expenses brought by NICU admittance. An infant whose postconceptual age is <46 weeks should not be transferred to ICU routinely after surgery, when their body weight exceeds 3.15 kg. Moreover for those with a body weight <3.15 kg, no matter what his/her postconceptual age is, medical personnel and parents should be warned of the possibility of the occurrence of postoperative apnea, desaturation and consequential NICU admittance.

Furthermore, we did not witness the beneficial effect of dexamethasone, maybe because laryngospasm was too rare (3/267) in this study in contrast to a beneficial effect of steroids in one-lung ventilation children (22). Use of muscle relaxant or opioids did not increase the prevalence of desaturation or ICU admittance, which is maybe due to minimizing doses, giving muscle relaxant antagonist at the end of surgery and our attempt to achieve opioid-sparing regimen.

There are several limitations of this study. Firstly, after dimension deduction, there are 6 potential risk factors, almost each of which were combinations of several observational variables. Thereafter, when converting to measurable regression equation, confidence interval was enlarged, which conveyed a low accuracy. Even though the combined risk factors could predict postoperative complications more precisely, we calculated the odd ratio for each observational variable in order to apply the results in clinical guidance. Secondly, collinearity test showed that there was no multicollinearity, but the confidence interval was enlarged, which could be caused by small sample size in this study. Thirdly, despite of the continuous variables determined as risk factors for different complications, a cut-off point should be ideally ascertained. In this scenario, RR value was based on univariate analysis, because almost no more significant factor was found in the regression models. Finally, this is not an interventional study, which could not eliminate bias or confounding factors. The advantage of less invasive airway, LMA was not examined in our study, which would be studied in the future.

In conclusion, this study found that preoperative atropine and intubation would prevent bradycardia and low body weight is a predictor for both bradycardia and postoperative desaturation in preterm infants undergoing ROP surgeries.

## Data Availability Statement

All datasets generated for this study are included in the article/supplementary material.

## Ethics Statement

The studies involving human participants were reviewed and approved by Peking University People's Hospital Institutional Review Board. Written informed consent from the participants' legal guardian/next of kin was not required to participate in this study in accordance with the national legislation and the institutional requirements.

## Author Contributions

BJ: data analysis. LY, YF, and HZ: study design, patient recruitment, data collection and writing of the paper. JL: Patient recruitment and data collection.

## Conflict of Interest

The authors declare that the research was conducted in the absence of any commercial or financial relationships that could be construed as a potential conflict of interest.
